# Psychometric properties of the Persian version of the COVID-19 Phobia Scale (C19P-S)

**DOI:** 10.1186/s12888-022-04507-9

**Published:** 2023-01-04

**Authors:** Razieh Bandari, Majideh Heravi-Karimooi, Shaahin Khosravi, Peghahsadat Yosefi, Mehri Omidian, Ali Montazeri

**Affiliations:** 1 Social Determinants of Health Research Center, Semnan University of Medical Sciences, Semnan, Iran; 2 Elderly Care Research Center, Faculty of Nursing & Midwifery, Shahed University, Tehran, Iran; 3 Kowsar Hospital, Semnan University of Medical Sciences, Semnan, Iran; 4 Department of Operation Room and Anesthesiology, Sorkheh School of Allied Medical Sciences, Semnan University of Medical Sciences, Semnan, Iran; 5 Population Health Research Group, Health Metrics Research Centre, Iranian Institute for Health Sciences Research, ACECR, Tehran, Iran; 6 Faculty of Humanity Sciences, University of Science &Culture, Tehran, Iran

**Keywords:** Coronavirus, COVID-19, Phobia, C19P-S, Validity, Psychometric, Reliability

## Abstract

**Background:**

During various infectious pandemics, phobia or panic has been suggested as one of the most common mental disorders. The current study reports on the psychometric properties of the Persian version of the COVID‐19 Phobia Scale (C19P-S) in Iran.

**Methods:**

The forward–backward translation procedure was applied to translate the English version of the C19P-S into Persian. Then, content and face validity, structural validity (exploratory and confirmatory factor analyses), convergent and discriminant validity, concurrent validity, reliability, and stability were performed to evaluate the Persian version.

**Results:**

In all, 660 people participated in the study. The mean age of patients was 35.55 (SD = 12.24) years. Exploratory factor analysis confirmed a four-factor structure for the scale. Confirmatory factor analysis showed that almost all fitness indices for the model were satisfactory (RMSEA = 0.06, CFI = 0.96, TLI = 0.96, IFI = 0.97). The Cronbach’s alpha coefficient and intraclass correlation coefficient (ICC) for the questionnaire were 0.95 and 0.96, respectively.

**Conclusion:**

The Persian version of C19P-S showed good psychometric properties and a good fit for the four-factor structure. It can now be used to assess panic disorder in therapeutic settings and identify candidates needing clinical intervention.

**Supplementary Information:**

The online version contains supplementary material available at 10.1186/s12888-022-04507-9.

## Background

The Coronavirus (COVID-19) appeared in Wuhan, China, in January 2020 and quickly spread to many countries. The mortality rate for COVID-19 is declining, but the disease is not yet completely dormant. Covid-19 has strongly influenced various aspects of daily life, including political, social, occupational, psychological, and economic. Signs and symptoms of COVID-19 include shortness of breath, dry cough, fatigue, and fever. After repeated fluctuations in mortality, the mortality rate has stabilized at about 4 to 11 percent. Iran has the highest pollution level in the Middle East and is in a dangerous situation due to medical shortages [1–3].

People usually experience anxiety symptoms such as anxiety, phobia, and fear after various infectious disease outbreaks. For example, in pandemics such as Ebola and Zika, the incidence of anxiety and panic disorders multiplied in a short period [4, 5].

A study showed that the prevalence of generalized anxiety disorder in the Iranian population is more than 27% [6]. In another survey of the Iranian people, the results showed that most participants experienced normal levels of stress (36.6%), anxiety (57.9%), and depression (47.9%). About 2.5% of respondents report very severe levels of stress. Also, the pathological levels of anxiety and depression were 6.3 and 7.9%, respectively [7].

Extensive research has shown that phobia is the most common psychiatric symptom worldwide. Phobia is an anxiety disorder defined as a persistent and intense fear of a particular situation or object. Phobias are classified into market phobia, social anxiety, and specific phobias. Specific phobias are broadly classified as animal phobias, nature phobias, environmental phobias, blood phobias, ampule phobias, injury phobias, situational phobias, and other phobias [8].

Significant research has been done on the prevalence of phobias during the corona pandemic. Any natural or human catastrophe can be considered a specific phobia. As epidemics such as COVID-19 have spread worldwide and people’s regular activities have been severely disrupted, the virus (Covid-19) was not immune to this [9]. The emotional and behavioral parts of the human psyche have been severely affected by the outbreak of COVID-19, and this new psychological distress can be cited as a particular phobia. Specific phobias can trigger other anxiety disorders associated with suicidal ideation, major depression, anxiety disorders, and physical, mental, or mood disorders [10]. Research has also shown that COVID-19 phobia disorder is highly prevalent among children, adolescents, adults, the elderly, people with pre-existing psychiatric conditions, and the front-line treatment staff for the virus. Among the causes of the phobia of the virus is the lack of information about COVID-19, the lack of public vaccination, occupational problems due to quarantine, and the ambiguity of returning to everyday life [11]. Therefore, various medicine and psychology clinicians must consider this type of phobia.

Now we are in the post-pandemic era a time that psychological distress is very common. One of the most deserving disorders is stress and phobia. Indeed, finding the cause of such symptoms is more important than trying to deal with the symptoms alone. Otherwise, the symptoms will recur and people constantly might experience a condition so-called a state of danger [12]. Thus, assessing phobia in this period seems essential. Recently a self-report questionnaire with 20 questions about coronaphobia was developed and showed promising results in recognizing coronaphobia in psychological, psychosomatic, social, and economic dimensions [13]. As such we have decided to translate and validate the instrument in Iran.

## Methods

### The COVID‐19 Phobia Scale (C19P‐S)

The C19P‐S Contains 20 items and is used to assess the level of coronaphobia. All items in this questionnaire are graded on a 5-point Likert scale from strongly disagree = 1 to strongly agree = 5. The range of scores varies between 20 and 100. This questionnaire includes four psychological, psychosomatic, social, and economic subscales. The psychological subscale is measured by the first six items, the psychosomatic subscale by items 7 to 11, the social subscale by items 12 to 15, and the economic subscale by items 16 to 20. The total score of Cronbach's alpha questionnaire was reported as 0.80 and 0.92 [13].

### Translation procedure

After obtaining permission from the author (dated January 11, 2021, signed by Mustafa Baloglu), a forward–backward translation procedure was applied. The English version was translated into Persian by two independent professionals. Then a single forward Persian version was provided by the research team. Consequently, two professionals back-translated the Persian version into English. Again the research team provided a single backward English version of the questionnaire and compared it with the original version. Since we did not observe any differences thus a provisional Persian version of the questionnaire was subjected to content and face validity as described below:

#### Content validity (qualitative content validity)

Fifteen experts (three clinical psychologists, three psychiatrists, five assistant professors of nursing, and four assistant professors with experience in questionnaire design) were asked to review the questionnaire qualitatively and offer their opinions on its accuracy, grammar, vocabulary, word placement, and response categories to assess its content validity.The experts did not alter the questionnaire in any way.

#### Face validity (qualitative face validity)

To evaluate the face validity of the questionnaire, the C19P-S was given to 10 individuals who met the inclusion criteria with the maximum variance. Their opinions regarding the items' appropriateness, difficulty, and ambiguity were evaluated. Almost all respondents did not mention any problems; thus, the questionnaire was prepared for psychometric analysis (Additional file 1).

#### Psychometric evaluation

This was a cross-sectional study. For the study purposes, we thought at least 200 people (10 participants per item) are needed for exploratory factor analysis (EFA), and similarly, 200 people are needed for confirmatory factor analysis (CFA). However, in practice, overall, we recruited 660 people living in Semnan, Iran. All participants were asked to complete the study questionnaires in a calm setting. In the case of less educated individuals, the principal investigator (PY), (SHKH), and (MO) helped people to complete the questionnaires. In all instances, the completion of the questionnaires took about 15 min. The inclusion criteria were as follows: being 18 years of age or older, willing to participate, being Iranian, and having no difficulty speaking or listening. Since several samples recruited via the online procedure, the questionnaire and consent form were uploaded to the https://porsline.ir Platform. Then the link was shared on Telegram, WhatsApp, and email groups of people.

#### Additional measures

1. All people were asked to respond to a short demographic questionnaire including items ongender, age, educational attainment, marital status, employment status, number of children, living conditions, economic status, health status, chronic disease, COVID-19 diagnosis, and relative/friend dead from COVID-19.
2. COVID Stress Scales (CSS): This is a 36-item scale for assessing COVID-19 stress. The CSS consists of fivefactorsincluding fear about economic consequences, danger, contamination, compulsive checking and reassurance-seeking xenophobia, and traumatic stress symptoms related to COVID-19. The original version of the CSS showed suitable psychometric features in American and Canadian populations [14] The psychometric properties of the Persian COVID stress scale (Persian-CSS) showed satisfactory results [15].

### Statistical analysis

Several statistical analyses were carried out to assess the psychometric properties of the Persian version of the COVID-19 phobia scale (C19P-S) as follows:

### Construct validity

#### Structural validity

The Structural validity of the scale was evaluated using maximumlikelihood with Promax rotation exploratory factor analysis [16]. In the initial step, the latent factors were extracted based on Horn’s parallel analysis [17]. Then the Kaiser–Meyer–Olkin (KMO) test of sampling adequacy and the Bartlett test were implemented. The Bartlett Test of sphericity was utilized in the sample to confirm that the matrix underlying the correlational analysis is not zero. Values above 0.7 in the KMO test and p-values less than 0.05 in Bartlett's test were thought-about because of the quality criterion for correlational analysis [18]. The number of factors was determined based on Parallel Analysis and the scree plot.

Structural equation modeling with confirmatory factor analysis was used to test the relationships between variables and the instrument's psychometric properties. Model fit was evaluated using AMOS 23, and several indices were employed to measure the usefulness of the model. The following requirements need to be met: the chi-square statistic (χ2), chi-square ratio, and degrees of freedom (χ2 / df). Goodness-of-fit index (GFI), adjusted goodness-of-fit index (AGFI), mean square root approximate error (RMSEA), and CFI > 0.9, χ2 / d < 5, GFI > 0.9, and RMSEA < 0.08 are considered as appropriate indices and reasonable values. The second-order factor analysis assumed that the extracted latent variables within the initial stage were present. Thus, the second-order factor analysis depicted the additional general concepts at secondary and higher levels [19–21].

#### Convergent validity

Convergent validity is the assessment to measure the level of correlation of multiple indicators of thesame construct that are in agreement. To establish convergent validity, the factor loading of theindicator, construct reliability (CR), and the average variance extracted (AVE) have to be considered (22) [22]. The value ranges from 0 to 1. AVE value should exceed 0.50 so that it is adequate for convergentvalidity [22–25].

#### Discriminant validity

Discriminant validity is referring to the extent to which the construct is differing from oneanother empirically. It also measures the degree of differences between the overlapping construct [22]. The discriminant validity can be evaluated by using cross-loading of indicator, Heterotrait-monotrait (HTMT) ratio of correlation [26]. HTMT valuesclose to 1 indicate a lack of discriminant validity. Using the HTMT as a criterion involves comparingit to a predefined threshold. If the value of the HTMT is higher than this threshold, one can concludethat there is a lack of discriminant validity. Some authors suggest a threshold of 0.85 [27]. In addition,Gold et al. argued with it and proposed a value of 0.90 [24, 28].

#### Concurrent validity

**Fig. 1:**
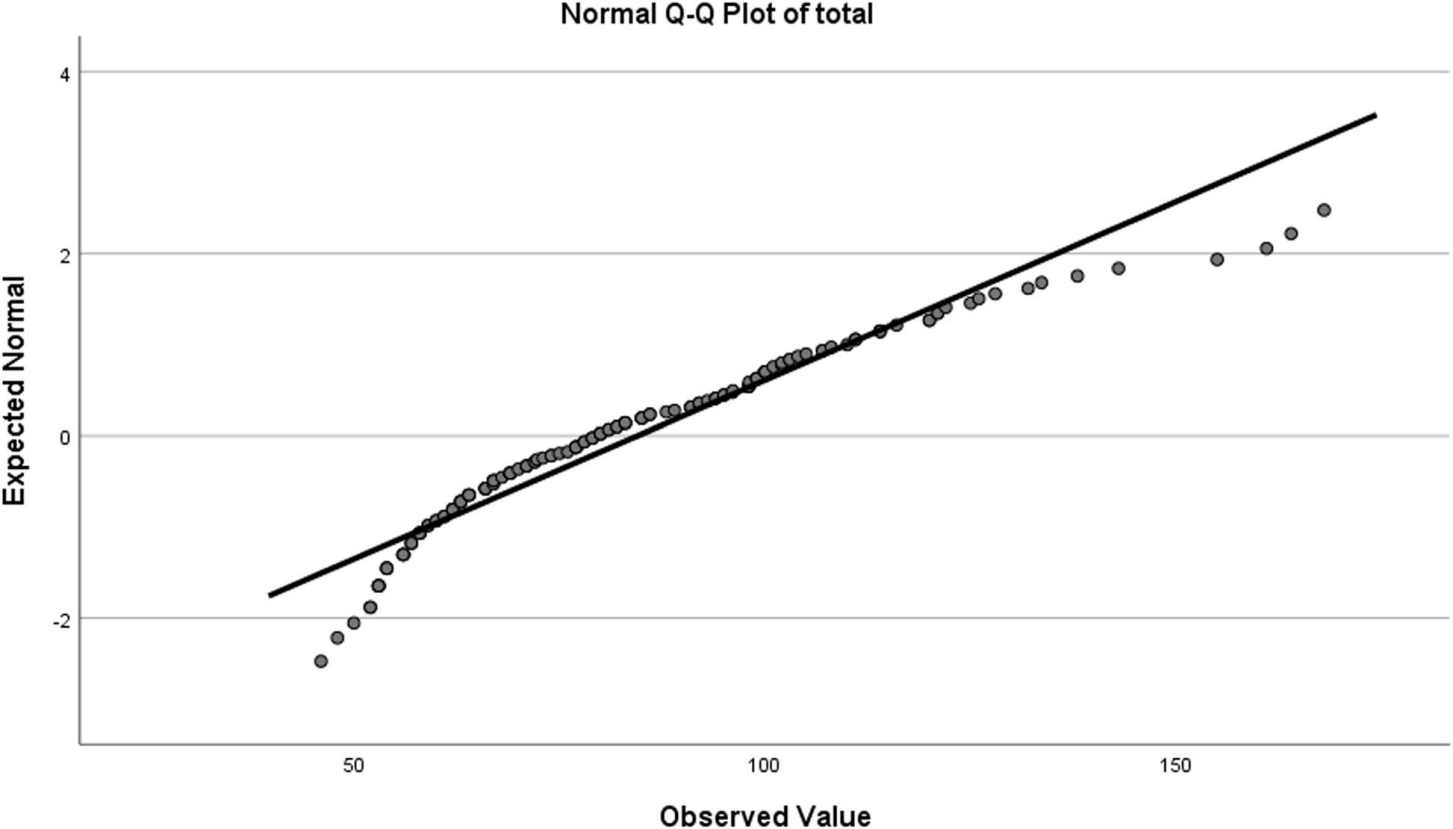
Q-Q Plot Scree

First, the normality of the data was evaluated using the Kolmogorov–Smirnov (K-S) test,the Shapiro–Wilk (S-W) test, and the QQ plots. Since the findings showed that the data was not normally distributed (Fig. 1), the concurrent validity was assessed by correlation between the C19P-S and the COVID Stress Scales (CSS) using the Spearman’s correlation coefficient.

### Reliability

#### The Cronbach’s alpha and McDonald’s omega

The Cronbach’s alpha and McDonald’s omega were used to investigate the internal consistency of the questionnaire [29, 30]. Then, the Construct Reliability (CR) was calculated [31]. The internal consistency of the scale was considered to be appropriate if it was equal to or greater than 0.7, and CR greater than 0.7 was considered to be acceptable [32, 33].

#### Stability

A sub-sample of 50 people completed the questionnaire twice at a two-week interval. The sub-sample was drawn randomly from the original sample consisting of 28 females and 22 males. To test stability the intraclass correlation coefficient (ICC) using two-way mixed ICC for absolute agreement at individual item level was performed [19, 33].

### Normal distribution, outliers, and missing data

The normal distribution of the data, the outliers, and the missing data were separately assessed. The distribution of data was investigated by skewness (± 3) and kurtosis (± 7). The presence of outliers was assessed using the Mahalanobis d-squared method (p < 0.001) and normality by the Mardia coefficient of multivariate kurtosis (> 8). Fortunately, there were no missing data. Data analysis was carried out using SPSS-AMOS 24, Jamovi-2.3.18.0, and JASP-0.16.4.

## Results

### Participants

Overall, 660 people participated in the study. Three hundred people viewedthe survey via an online approach. Of these, 220 people completed the questionnaires giving a response rate of 73%. This sample was used for confirmatory factor analysis. The remaining samples were recruited in health centers (440). The response rate for this group was 100%.Participants' demographic data are given in Table 1. As shown the mean age (SD) of participants was 35.55 (12.24) years; 451 were women (68.3%), and 49.4% were married.

**Table 1:** The characteristics of study participants (*n* = 660)

		**Exploratory sample (*n* = 440)**	**Confirmatory sample (*n* = 220)**	**(%)**
**Gender**				
	Man	140	69	31.7
	Female	300	151	68.3
**Age groups (years)**				
	< 40	53	26	11.9
	41–60	239	119	54.2
	> 60	148	75	33.9
**Mean (SD)**	35.55 (12.24)	-	-	-
**Educational**				
	Less educated	54	27	12.3
	Primary	141	71	32.1
	Secondary	95	47	1.5
	Higher	150	75	34.1
**Marital status**				
	Married	217	109	49.4
	Single	215	107	48.8
	Widowed	5	3	1.2
	Divorced	3	1	0.6
**Employment status**				
	Housewife	76	38	17.3
	Employed	318	159	72.3
	Retired	35	17	7.9
	Unemployed	11	6	2.5
**Number of children**				
	0	79	39	7.9
	1–3	201	101	45.8
	4–6	144	72	32.7
	> 7	16	8	3.6
**Living condition**				
	Alone	70	35	15.9
	with spouse	129	64	29.2
	With children	119	59	27.0
	With his spouse and children	113	56	25.6
	Others	10	5	2.3
**Economic status**				
	Poor	111	55	25.3
	Intermediate	185	93	42.1
	Good	144	72	32.7
**Housing**				
	The owner	335	168	76.2
	Tenant	95	47	21.5
	Children's home	7	3	1.5
	Familiar home	3	2	0.8
**Health status**				
	Very Poor/poor	28	14	6.4
	Fair	146	73	33.2
	Good/very good	266	133	60.5
**Chronic disease**				
	Yes	166	82	37.6
	No	274	138	62.4
**COVID-19 diagnosis**				
	Positive	72	35	16.2
	Negative	368	185	83.8
**Death of a relative/friend from COVID-19**				
	Yes	106	53	24.1
	No	334	167	75.9

### Exploratory factor analysis

**Fig. 2:**
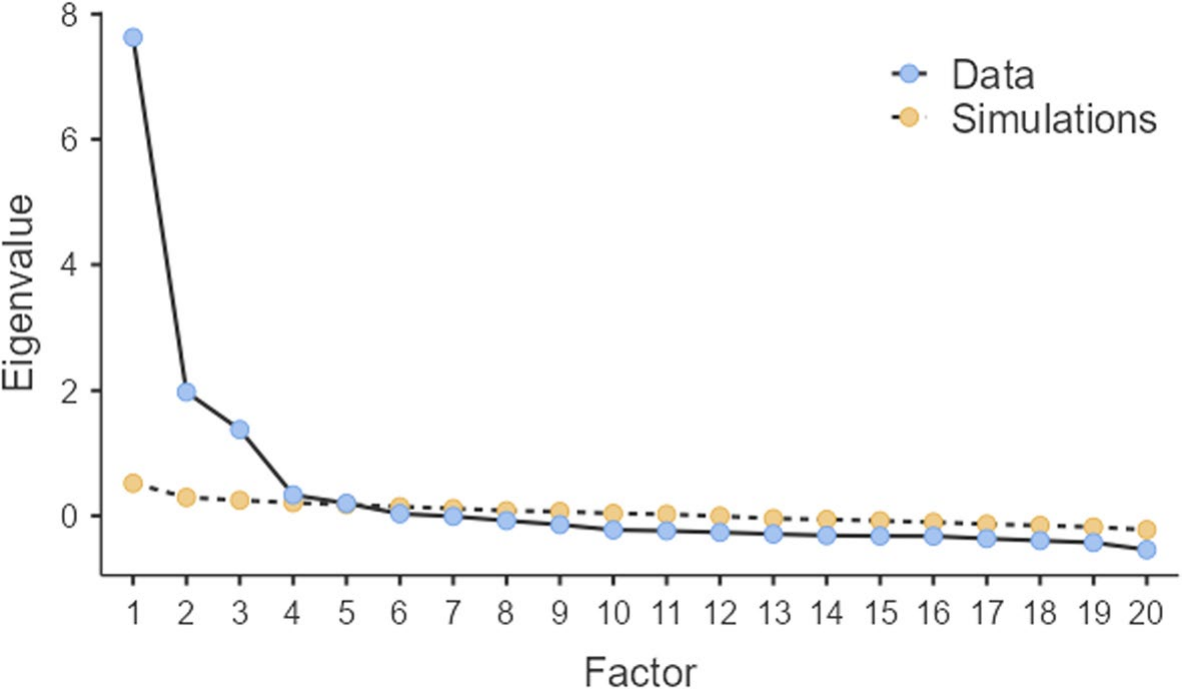
Plot for the Exploratory Factor Analysis (EFA) COVID-19 Phobia Scale (C19P-S)

The KMO statistic, indicating sampling adequacy, was (0.967). The sphericity test by Bartlett was statistically significant (X2 = 5549.245, P.0001). three items (items 9, 10,and 20) showed, as the values of communalities were < 0.2 and factor loadings were < 0.3. However, in the following analysis, four factors with eigenvalues > 1 were identified based on the factor analysis (Table 2), which explained 65.15% of the variance observed. The details of the maximum likelihood EFA results are shown in Table 2 and Fig. 2.

**Table 2:** Exploratory factor analysis of the COVID-19 Phobia Scale (C19P-S) (*n* = 440)

Items	F 1	F 2	F3	F4
1. The fear of coming down with coronavirus makes me very anxious	**0.912**	0.020	0.088	0.181
5. I am extremely afraid that someone in my family might become infected by the coronavirus	**.0863**	0.061	0.137	-.072
13. Uncertainties surrounding coronavirus cause me enormous anxiety	**0.841**	-0.062	0.108	0.232
17. The pace that coronavirus has spread causes me great panic	**0.776**	0.163	0.020	0.134
9. News about coronavirus-related deaths causes me great anxiety	**0.239**	0.144	0.138	0.029
20. I argue passionately (or want to argue) with people I consider to be behaving irresponsibly in the face of coronavirus	**0.294**	0.033	0.138	0.048
10.I experience tremors due to the fear of coronavirus	**0.104**	-0.065	0.128	0.020
6. I experience serious chest pain out of the fear of coronavirus	0.115	**0.861**	0.254	.065
14. I experience sleep problems out of the fear of coronavirus	0.000	**0.792**	-.004	0.005
18. Coronavirus makes me so tense that I find myself unable to do the thing I previously had no problem doing	-0.106	**0.768**	-.037	0.241
3. After the coronavirus pandemic, I feel extremely anxious when I see people coughing	-0.018	-0.037	**0.982**	0.028
7. After the coronavirus pandemic, I actively avoid people I see sneezing	0.053	0.012	**0.949**	0.153
11. Following the coronavirus pandemic, I have noticed that I spend extensive periods of time cleaning my hands	0.041	0.156	**0.740**	0.049
15. The fear of coming down with coronavirus seriously impedes my social relationships	0.057	0.202	**0.698**	0.006
19. I am unable to curb my anxiety of catching coronavirus from others	0.040	0.072	**0.668**	0.019
4. The possibility of food supply shortage due to the coronavirus pandemic causes me anxiety	0.124	-0.172	0.115	**0.929**
8. The possibility of shortages in cleaning supplies due to the corona virus pandemic causes me anxiety	0.012	0.126	0.198	**0.920**
12. I stock food with the fear of coronavirus	0.131	0.033	0.012	**0.757**
16. After the coronavirus pandemic, I do not feel relaxed unless I constantly check on my supplies at home	0.172	0.136	0.045	**0.745**
*Eigenvalue*	**6.59**	**7.00**	**7.22**	**6.91**
*% Variance*	**41.33**	**10.61**	**7.56**	**5.65**

F1: Psychological, F2: Psycho-somatic, F3: Social, F4: Economic

### Confirmatory factor analysis

**Fig. 3:**
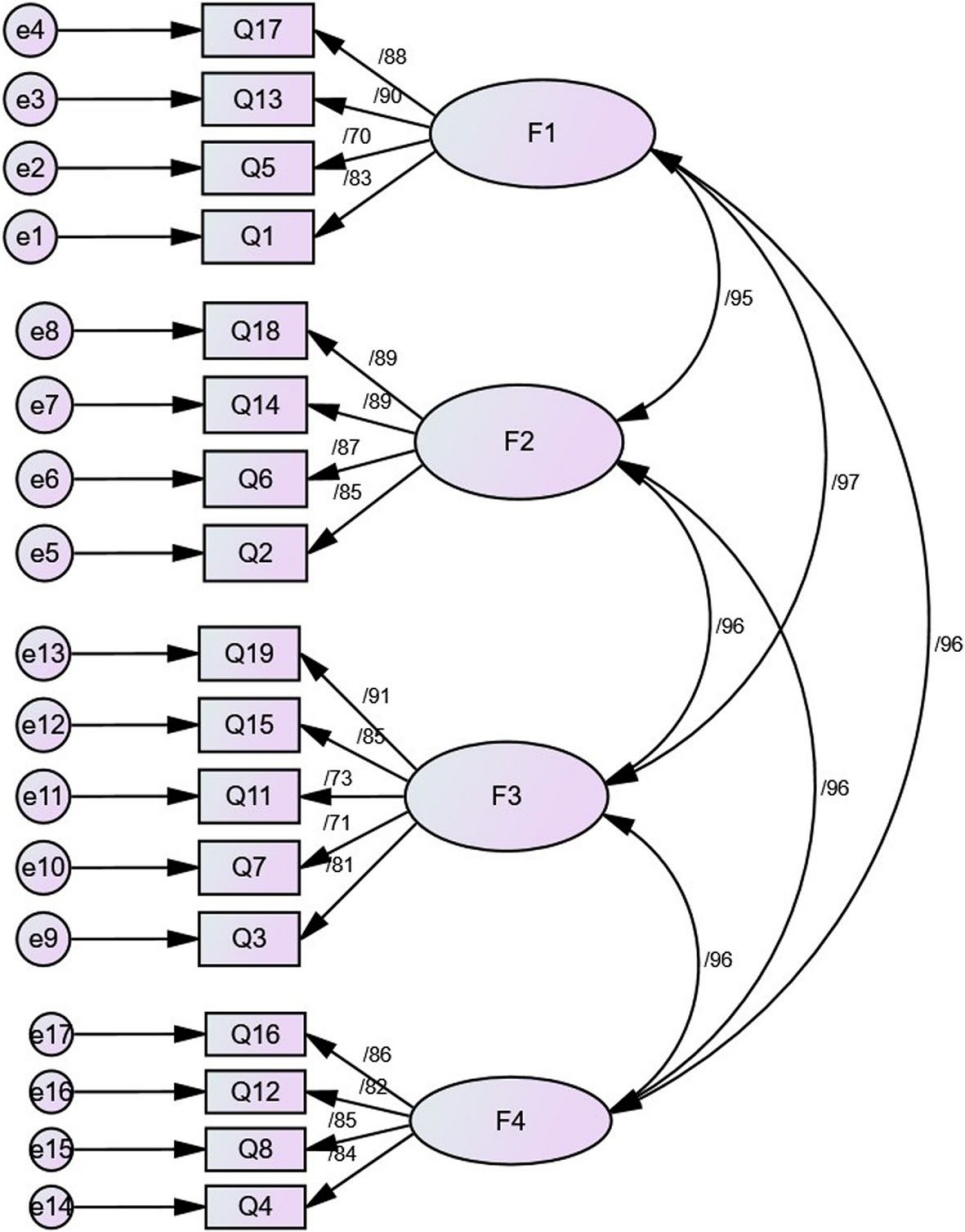
First-order confirmatory factor analysis

**Fig. 4:**
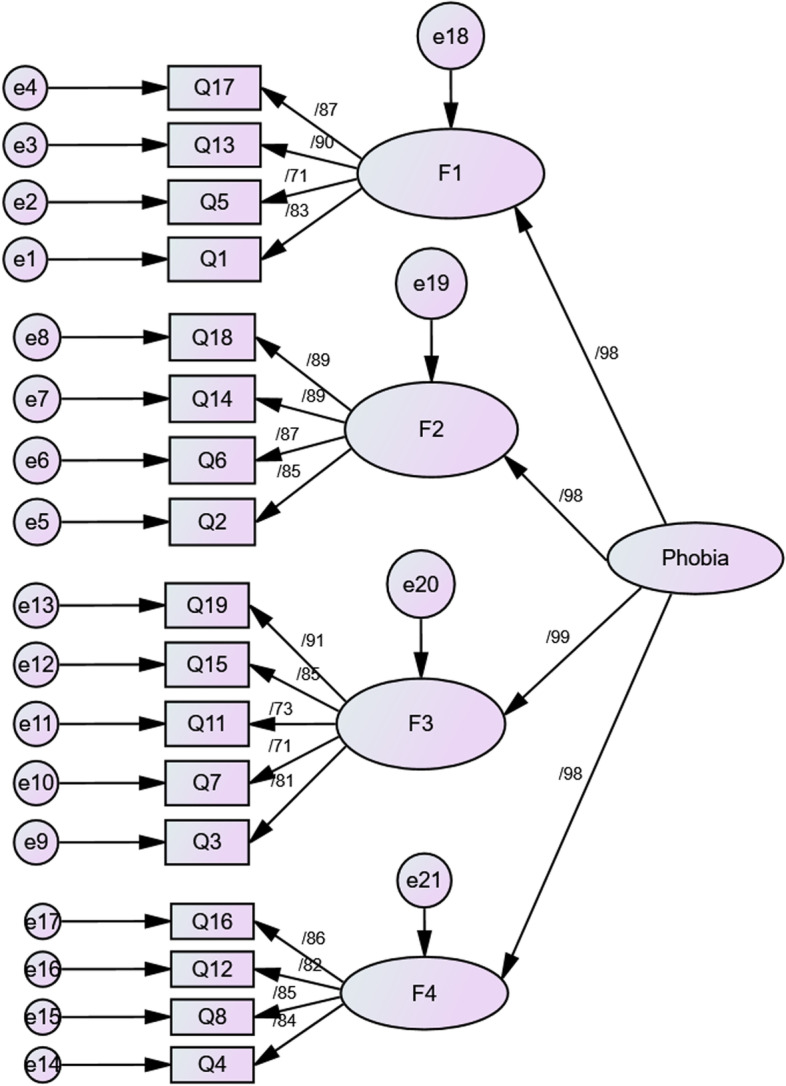
Second-order confirmatory factor analysis

We conducted CFA to examine the suitability of the hypothetical three-factor structure of the C19P-S. Table 3 presents the goodness of fit values of the testing models. Overall, the three-factor model of the C19P-S was confirmed. Following the first-order CFA, the high correlation between the dimensions indicated that a single factor was associated with all dimensions (Fig. 3). Thus, second-order factor analysis was performed to examine whether all factors were contributed to by the common factor of the C19P-S (Fig. 4).

**Table 3:** Goodness of fit indexes results from confirmatory factor analysis (*n* = 220)

Indexes*	Cut off values	First order	Second order
CMIN/df	< 3	1.929	1.929
*P* value**	≥ 0.05	0.0001	0.0001
RFI	≥ 0.90	0.91	0.91
TLI	≥ 0.95	0.96	0.96
IFI	≥ 0.90	0.96	0.96
CFI	≥ 0.95	0.96	0.96
PCFI	≥ 0.5	0.83	0.83
PNFI	≥ 0.5	0.79	0.79
RMSEA	≤ 0.08	0.06	0.06

^*^ *Abbreviations*: *CMIN/DF* Minimum Discrepanscy Funcation by degrees of freedom divided, *RFI* Relative Fit Index, *TLI* Tucker –Lewis Index, *IFI* Incremental Fit Index, *CFI* Comparative Fit Index, *PCFI* Parsimonious Comparative Fit Index, *PNFI* Parsimonious Normed Fit Index, *RMSEA* Root Mean Square Error of Approximation

^**^ Derived from chi-square test (χ^2^), which is one of the measures for fit indexes in structural equation modeling (SEM). A non-significant result for this test indicates a good model fit

### Convergent validity

Output from the analysis revealed the construct reliability (CR), the average variance extracted (AVE), and the correlation coefficients between the constructs that are summarized as in Table 4.

**Table 4:** Construct reliability (CR), the square root of the average variance extracted (AVE), and correlations between constructs (off-diagonal)

	**F1**	**F2**	**F3**	**F4**	**CR**	**AVE**	**MSV**	**MaxR(H)**
**F1**	0.752				0.883	0.565	0.934	0.922
**F2**	0.956***	0.814			0.904	0.662	0.930	0.932
**F3**	0.967***	0.964***	0.805		0.901	0.648	0.934	0.920
**F4**	0.957***	0.961***	0.964***	0.842	0.907	0.709	0.928	0.908

*MSV* Maximum Shared Variance, *AVE* Average variance extracted, *CR* Construct Reliability, M*axR (H)* Maximum Reliability

F1: Psychological, F2: Psycho-somatic, F3: Social, F4: Economic

Significance of Correlations: *** *p* < 0.001

The CR for all constructs are above 0.70 and the AVE values are between 0.565 and0.709. The convergent validity was assessed using Fornel and Larcker (1971) by comparing the squareroot of each AVE in the diagonal with the correlation coefficients (off-diagonal) for each construct inthe relevant rows and columns. Overall, convergent validity can be accepted for thismeasurement model and supports the convergent validity between the constructs.

### Discriminant validity: Heterotrait-monotrait (HTMT) criterion

Since the HTMT results, the values in Table 5 indicated discriminant validity problemsaccording to the HTMT_0.85_criteria. This implied that the HTMT criterion detects the collinearity problems among the latent constructs.Probably most of the items of constructs are measuring the same thing. In other words, itcontains the overlapping items from the respondents’ perceptionsof the affected constructs.

**Table 5:** Heterotrait-monotrait (HTMT) ratio of correlation

	Psycho-somatic	Social	Economic	Psychological
Psycho-somatic	1			
Social	0.947	1		
Economic	0.969	0.965	1	
Psychological	0.979	0.960	0.958	1

F1: Psychological, F2: Psycho-somatic, F3: Social, F4: Economic

### Concurrent validity

The Spearman’s correlation coefficient between the COVID-19 phobia scale (C19P-S) and the COVID Stress Scales was significant (*r* = 0.69, *p* < 0.05) (Table 6). There were positive and significant correlations between the measures. This confirmed that individuals with high levels of coronaphobia also tended to have higher anxiety levels.

**Table 6:** The correlation between the C19P-S and CSS

	**CSS**	**Psychological**	**Psycho-somatic**	**Social**	**Economic**	**Total C19P-S**
CSS	1					
Psychological	0.65^**^	1				
Psycho-somatic	0.60^*^	0.71^*^	1			
Social	0.64^**^	0.87^**^	0.89^*^	1		
Economic	0.63*	0.83*	0.86*	0.84*	1	
Total C19P-S	0.69^*^	0.95^*^	0.78^*^	0.60^*^	0.86*	1

^**^Correlation is significant at the *p* < 0.01 level (2-tailed)

^*^Correlation is significant at the *p* < 0.05 level (2-tailed)

### Reliability

Internal consistency of all items in the C19P-S scale was calculated using Cronbach’s alpha (0.84, 0.87, 0.86, and 0.87) and McDonald’s Omega as well as, which yielded (0.85,0.88,0.86, and 0.87), respectively. The intra-class correlation coefficient (ICC) for C19P-S and its subscales was higher than 0.7, indicating an acceptable agreement between test–retest scores. The results are shown in Table 7.

**Table 7:** The Cronbach's alpha, McDonald Omega coefficient (ω), and the Intraclass Correlation Coefficients (ICC) for the COVID-19 Phobia Scale (C19P-S)

	**Number of items**	**Cronbach’s alpha (*n* = 440)**	**McDonald's (ω)**	**ICC (n = 50)** **[CI = 95%]**	**P**
Psychological	7	0.84	0.85	0.82[0.67–0.91]	< 0.0001
Psycho-somatic	4	0.87	0.88	0.82[0.67–0.91]	< 0.0001
Economic	5	0.86	0.86	0.85[0.65–0.93]	< 0.0001
Social	4	0.87	0.87	0.85[0.71–0.92]	< 0.0001
Total	20	0.95	0.95	0.96[0.93 –0.96]	< 0.0001

*ICC* Intraclass correlation coefficient

## Discussion

Evaluation of psychometric properties of the C19P-S in a sample of Iranian population showed that it is a valid and reliable instrument for assessing COVID-19 phobia.The findings from the current study indicated a four-factor structure for the Persian version of the questionnaire, including 20items that tapped into psychological (7 items), psycho-somatic (4 items) economic (5 items), and social (4 items) factors.

Psychological symptoms, including worry, panic, and phobia, are common during the pandemic. It is argued that such psychological symptoms might be due to long-term quarantine, false information about the virus, posttraumatic stress disorder, confusion, and anger [34].

A study found that psychological factors were the most prevalent COVID-19 phobia determinants.They also emphasized the significance of psychosomatic elements in explaining covid-19 phobia, which is linked to physiologic difficulties and psychosocial concerns. The COVID-19 pandemic is just one example of a stressful life event that has been related in the past to persistent pain and gastrointestinal issues [35].

Because of the global economic and social disruption caused by the COVID-19 pandemic, has negatively impacted people's mental health [36]. Previous research indicated that the harmful impacts of the COVID-19 pandemic extended beyond just psychological ones, including economic and social impacts [37]. This is supported by the finding of a recent study that COVID-19 may indirectly cause global unemployment and inflation to rise [38].

Social interactions have undergone profound alterations as a result of the pandemic [39]. Previous research has shown that the COVID-19 pandemic has caused excessive anxiety in the neighborhood; as a result, individuals attempt to keep their distance from one another in the streets and public areas to prevent spreading the virus to their families. Consistent with one study, the pandemic has had a significant negative influence on social communication. For instance, 76% of interviewees said they only spoke to family members during the pandemic [1].

The four-factor structure of the original C19P-S was validated by the CFA findings in the current investigation. These outcomes were comparable to the initial C19P-S. This study, therefore, demonstrated that COVID-19 can cause phobia in the population as psychological, psycho-somatic, economic, and social [9, 13, 40–45].

The results of this study showed that the items in the final model of the C19P-S had convergent validity [44, 45]. According to Hair et al., convergent validity exists when construct items are closely related and have high variance [46]. However, the discriminant validity as assessed by HTMT analysis was not confirmed. Also, in the original study, convergent validity was investigated through construct reliability (CR) and average variance extracted (AVE) values, as well as discriminant validity through the square root of AVE values [13].

Concurrent validityshowed that the questionnaire had a positive and significant correlation with the COVID stress scale indicating the appropriate validity of the COVID-19 phobia questionnaire. This finding is similar to previous results, according to which specific anxiety is often associated with different types of phobias [47, 48].

The present study examined internal consistency (through Cronbach's alphas and Omega coefficients) and test–retest reliability to demonstrate that the Persian-C19P-S had good stability (via ICC coefficients). For the C19P-S and all of its subscales, all Cronbach's alpha and Omega coefficients were higher than 0.70, demonstrating good reliability and similar to reported results of the original scale (0.95) [13], Korean (0.93) [41], Portuguese (0.92) [42], Turkish (0.92) [49], Indonesian (0.90) [43], Arabic (0.92) [40], United States (0.71–0.79) [9], European countries (0.93) [11], Nigeria (0.89) [44], and Japanese (90) [45]. Also, test–retest reliability coefficients calculated by ICC coefficients were higher than 0.70 for the scale and its subscales, confirming the good stability of the scale. These findings were in line with the original scale [13]. However, in the original study, only Cronbach’s alphas had been assessed, but the present study added new evidence in terms of confirming the stability of the scale [13].

### Limitations

This research, like other studies, has limitations. First, we did not collect the participants' medical and psychiatric history and thus this might be influenced the findings. The following limitation is that not everyone in the community may have access to the Internet, thus limiting the possibility of generalizing the results. However, due to the epidemic of coronaviruses, online implementation is a suitable method in the case of the COVID-19 pandemic.

### Implication

The current study offers psychologists and therapists to assess phobia. They also may develop targeted interventions to prevent the phobia generated by the pandemic.

## Conclusion

The present study proved that COVID-19 Phobia Scale (C19P-S) was a reliable and valid instrument to assess COVID-19 related phobia in Iran. To confirm the findings further studies might be warranted.

## Supplementary Information

**Additional file 1.** The Persian version of the COVID-19 Phobia Scale (C19P-S). 

**Additional file 2.** The Coronavirus 19 Phobia (CP19-S) Scale. 

## Data Availability

The datasets used and/or analyzed during the current study are available from the corresponding authors on reasonable request.

## Abbreviations

AVE: Average Variance Extracted
CFA: Confirmatory Factor Analysis
CFI: Comparative Fit Index
CMIN/DF: Minimum Discrepancy Function by degrees of freedom divided
CR: Construct Reliability
EFA: Exploratory Factor Analysis
GFI: Goodness of Fit Index
ICC: Intraclass Correlation Coefficients
IFI: Incremental Fit Index
KMO: Kaiser–Meyer–Olkin
MSV: Maximum Variance
PCFI: Parsimonious Comparative Fit Index
PNFI: Parsimonious Normed Fit Index
RMSEA: Root Mean Square Error of Approximation
SEM: Structural Equation Modeling
TLI: Tucker –Lewis Index
