# Supplementary material for: Psychometric properties of the Persian version of the COVID-19 Phobia Scale (C19P-S)
Source: BMC Psychiatry. 2023 Jan 4;23:8. doi: 10.1186/s12888-022-04507-9 (PMC9811708; doi:10.1186/s12888-022-04507-9)
Supplement: Supplementary file 1 — Additional file 1. The Persian version of the COVID-19 Phobia Scale (C19P-S). [file 12888_2022_4507_MOESM1_ESM.docx]

COVID-19 Phobia Scale (C19P-S)

| **کاملا موافقم** | **موافقم** | **نظری ندارم** | **مخالفم** | **کاملا مخالفم** |  |
| --- | --- | --- | --- | --- | --- |
|  |  |  |  |  | **ترس از ابتلا به ویروس کرونا مرا بسیار مضطرب می­کند** |
|  |  |  |  |  | **من از ترس ویروس کرونا دچار دردهای جدی در معده م­ شوم** |
|  |  |  |  |  | **بعد از همه گیری ویروس کرونا ، وقتی می بینم که مردم سرفه می کنند ، اضطراب شدیدی پیدا می­کنم** |
|  |  |  |  |  | **احتمال کمبود تأمین مواد غذایی به دلیل همه گیری ویروس کرونا باعث اضطراب من می­شود** |
|  |  |  |  |  | **من به شدت می ترسم که کسی در خانواده من به ویروس کرونا آلوده شود.** |
|  |  |  |  |  | **من از ترس ویروس کرونا دچار درد جدی در قفسه سینه می­شوم** |
|  |  |  |  |  | **بعد از همه گیری ویروس کرونا ، من به طور جدی از افرادی که عطسه می کنند پرهیز می­کنم.** |
|  |  |  |  |  | **احتمال کمبود مواد تمیزکننده به دلیل همه گیری ویروس کرونا باعث اضطراب من می­شود** |
|  |  |  |  |  | **اخبار مربوط به مرگ ناشی از ویروس کرونا باعث اضطراب شدید من می­شود** |
|  |  |  |  |  | **من به دلیل ترس از ویروس کرونا دچار لرزش می­شوم** |
|  |  |  |  |  | **به دنبال شیوع ویروس کرونا ،من متوجه شده ام که مدت زمان طولانی به تمیز کردن دست های خود می­پردازم.** |
|  |  |  |  |  | من از ترس ویروس کرونا مواد غذایی ذخیره می­کنم. |
|  |  |  |  |  | **عدم اطمینان در مورد ویروس کرونا باعث اضطراب زیادی برای من­می شود** |
|  |  |  |  |  | **من از ترس ویروس کرونا دچار مشکلات خواب می­شوم.** |
|  |  |  |  |  | **ترس از ویروس کرونا به طور جدی روابط اجتماعی من را از بین می­برد.** |
|  |  |  |  |  | **بعد از همه گیری ویروس کرونا ،احساس آرامش نمی کنم مگر اینکه مرتباً وسایل خود را در خانه ضد عفونی کنم** |
|  |  |  |  |  | **سرعت انتشار ویروس کرونا باعث وحشت شدید من می­شود** |
|  |  |  |  |  | **ویروس کرونا مرا بسیار دچار تنش می کند به نحوی خود را قادر به انجام کاری که قبلا در انجام آن مشکل نداشتم، نمی­بینم** |
|  |  |  |  |  | **نمی توانم اضطراب خود را برای گرفتن ویروس کرونا از دیگران کنترل کنم** |
|  |  |  |  |  | **من با افرادی که فکر می کنم در مقابل ویروس کرونا رفتار غیرمسئولانه ای دارند ، با جدیت بحث می کنم** |
