# Supplementary material for: Psychometric properties of the Persian version of the COVID-19 Phobia Scale (C19P-S)
Source: BMC Psychiatry. 2023 Jan 4;23:8. doi: 10.1186/s12888-022-04507-9 (PMC9811708; doi:10.1186/s12888-022-04507-9)
Supplement: Supplementary file 2 — Additional file 2. The Coronavirus 19 Phobia (CP19-S) Scale. [file 12888_2022_4507_MOESM2_ESM.docx]

**Coronavirus 19 Phobia (CP19-S) Scale***

DIRECTIONS: Following the coronavirus pandemic, people have begun to experience some of the difficulties listed below. Please read each statement carefully but without spending too much time on it. Consider your own situation throughout **THE LAST WEEK INCLUDING TODAY** and rate your agreement with each statement by selecting the corresponding circle. Please complete all the statements to the best of your ability.

For example, if you “agree” with the statement “coronavirus makes me anxious” when you consider yourself during the last week including today then select the second circle.

 Strongly                 Generally Strongly Disagree          Disagree          Agree        Agree          Agree Coronavirus makes me anxious.                            ①                       ❷                      ③                         ④                           ⑤

| **1.** The fear of coming down with coronavirus makes me very anxious. | ① ② ③ ④ ⑤ |
| --- | --- |
| **2.** I experience serious stomachaches out of the fear of coronavirus. | ① ② ③ ④ ⑤ |
| **3.** After the coronavirus pandemic, I feel extremely anxious when I see people coughing. | ① ② ③ ④ ⑤ |
| **4.** The possibility of food supply shortage due to the coronavirus pandemic causes me anxiety. | ① ② ③ ④ ⑤ |
| **5.** I am extremely afraid that someone in my family might become infected by the coronavirus. | ① ② ③ ④ ⑤ |
| **6.** I experience serious chest pain out of the fear of coronavirus. | ① ② ③ ④ ⑤ |
| **7.** After the coronavirus pandemic, I actively avoid people I see sneezing. | ① ② ③ ④ ⑤ |
| **8.** The possibility of shortages in cleaning supplies due to the cornavirus pandemic causes me anxiety. | ① ② ③ ④ ⑤ |
| **9.** News about coronavirus-related deaths causes me great anxiety. | ① ② ③ ④ ⑤ |
| **10.** I experience tremors due to the fear of coronavirus. | ① ② ③ ④ ⑤ |
| **11.** Following the coronavirus pandemic, I have noticed that I spend extensive periods of time cleaning my hands. | ① ② ③ ④ ⑤ |
| **12.** I stock food with the fear of coronavirus. | ① ② ③ ④ ⑤ |
| **13.** Uncertainties surrounding coronavirus cause me enormous anxiety. | ① ② ③ ④ ⑤ |
| **14.** I experience sleep problems out of the fear of coronavirus. | ① ② ③ ④ ⑤ |
| **15.** The fear of coming down with coronavirus seriously impedes my social relationships. | ① ② ③ ④ ⑤ |
| **16.** After the coronavirus pandemic, I do not feel relaxed unless I constantly check on my supplies at home. | ① ② ③ ④ ⑤ |
| **17.** The pace that coronavirus has spread causes me great panic. | ① ② ③ ④ ⑤ |
| **18.** Coronavirus makes me so tense that I find myself unable to do the thing I previously had no problem doing. | ① ② ③ ④ ⑤ |
| **19.** I am unable to curb my anxiety of catching coronavirus from others. | ① ② ③ ④ ⑤ |
| **20.** I argue passionately (or want to argue) with people I consider to be behaving irresponsibly in the face of coronavirus. | ① ② ③ ④ ⑤ |
